# Applications of Machine Learning in Genomics and Systems Biology

**DOI:** 10.1155/2013/587492

**Published:** 2013-02-07

**Authors:** Chunmei Liu, Dongsheng Che, Xumin Liu, Yinglei Song

**Affiliations:** ^1^Department of Systems and Computer Science, Howard University, Washington, DC 20059, USA; ^2^Department of Computer Science, East Stroudsburg University, East Stroudsburg, PA 18301, USA; ^3^Department of Computer Science, Rochester Institute of Technology, Rochester, NY 14623, USA; ^4^Department of Mathematics and Computer Science, University of Maryland Princess Anne, Eastern Shore, MD 21853, USA

As the accomplishment of the human genome project, techniques that can analyze large amounts of data are urgently needed. Advances in computational techniques for analyzing high-throughput data in genomics, proteomics, and visualization have been extensively studied and have played vital roles in understanding biological mechanisms. Machine learning and related techniques such as support vector machines, Markov models, decision trees, and neural networks have been increasingly used to solve problems in genomics and systems biology.

Machine learning was defined as a “computer program that can learn from experience with respect to some class of tasks and performance measure” [1]. If we can design machine learning algorithms to learn from past experience and thus improve the performance automatically, we can solve complicated problems such as those in genomics and systems biology. 

In this special issue, we have explored the topics of identifying biomarkers, transcription factor binding, novel type III effectors, predicting breeding values for dairy cattle, and gene selection and tumor classification. The papers in this volume have studied the previously researched domains and also researched the new approaches for bioinformatics problems. The papers reflect the urgency of using machine learning techniques to develop more efficient and accurate algorithms for biological problems. We hope that the papers in the volume can broaden the view of the current machine learning approaches in genomics systems biology and inspire ideas of designing new approaches for existing biological problems.

*Chunmei Liu* *Dongsheng Che* *Xumin Liu* *Yinglei Song*
